# Circulating miRNAs in Serum as Biomarkers for Early Diagnosis of Non-small Cell Lung Cancer

**DOI:** 10.3389/fgene.2021.673926

**Published:** 2021-07-09

**Authors:** Xiaotong Duan, Simiao Qiao, Dianhe Li, Shangbiao Li, Zhihao Zheng, Qin Wang, Xiaoxia Zhu

**Affiliations:** Department of Radiation Oncology, Zhujiang Hospital, Southern Medical University, Guangzhou, China

**Keywords:** non-small cell lung cancer, healthy controls, microRNA, biomarkers, early diagnosis

## Abstract

**Background:**

Non-small cell lung cancer (NSCLC) accounts for about 85% of lung cancers. This study aimed to discover the potential miRNA biomarkers for early detection of NSCLC.

**Methods:**

Total circulating miRNAs were extracted from six patients and six volunteers and run on the miRNA chip. The differentially expressed miRNAs acquired by data mining were intersected with chip results, and qRT-PCR were carried out. Then the differentially miRNAs were validated by using a validation cohort (120 participants). ROC curves were established to evaluate the diagnostic efficacy of the differentially circulating miRNAs. The target genes of the differential miRNAs were identified using the miRTarBase database, and follow-up GO and KEGG enrichment analysis were conducted.

**Results:**

We identified 577 miRNA which screened according to the criteria (fold change > 2 and *p* value < 0.05). Among them, seven circulating miRNAs passed additional filtering based on data mining. These miRNAs were further validated in the training and validation cohort. miR-492, miR-590-3p, and miR-631 were differentially expressed in the patients’ serum, and the area under the ROC curve (AUC) values of these miRNAs were 0.789, 0.792, and 0.711, respectively. When using them as a combination to discriminate healthy volunteers from patients, the AUC reached 0.828 (95% CI, 0.750–0.905, *p* = 0.000) with a sensitivity of 86.7% and specificity of 71.7%. The follow-up enrichment analysis showed that target genes of three miRNA were associated with tumorigenesis and progression, such as cell cycle and P53 signaling pathway.

**Conclusions:**

The combination of miR-492, miR-590-3p, and miR-631 can be utilized to distinguish healthy individuals and early-stage NSCLC patients.

**Impact:**

The combination of miR-492, miR-590-3p, and miR-631 might be a promising serum biomarker in patients for the early diagnosis of NSCLC.

## Introduction

Lung cancer has become the leading cause of cancer-related death, in which non-small cell lung cancer (NSCLC) accounts for 85% (Chen et al., 2014; Siegel et al., 2020). Early stage (stage I and II) NSCLC patients can achieve a good prognosis after receiving treatments (Smolle-Juettner et al., 2010; Ghysen and Vansteenkiste, 2019). It was reported that the 5-year survival rate of NSCLC patients with stage I who received a lobectomy was around 45–65% (Ou et al., 2007), while for those patients who were inoperable or had refused surgical treatment, the local control rate of stereotactic radiotherapy could be over 85%, and the 3-year survival rate was about 60%(Baumann et al., 2009; Timmerman et al., 2010). Therefore, early and precise diagnosis of NSCLC is essential for reducing the mortality of NSCLC patients (Osmani et al., 2018). The clinical diagnosis of NSCLC is usually based on histopathological diagnosis, whereas the definitive results must be obtained by invasive examination, such as operation and biopsy, which are invasive and painful for the patients (Postmus et al., 2017; Duma et al., 2019). Owing to the limitation of detection technique and invasiveness of the existing histopathological examinations, molecular profiling by non-invasive detection of the tumor associated gene expression might play an important role.

Recently, microRNAs (miRNAs) are found to be closely associated with the development of cancer (Li et al., 2013; Tutar, 2014). As a non-coding single-stranded small RNA with 19-24 nucleotides, miRNAs mediate the post-transcriptional gene expression by binding to the 3’ non-coding region of the target gene (Lu and Rothenberg, 2018; Correia de Sousa et al., 2019). It is highly conservative and endogenous, and it participates in a majority of biological processes including cell proliferation, differentiation, apoptosis (Bartel, 2004; He and Hannon, 2004). More than 50% of miRNA genes are located in tumor-associated genomic regions (which were called fragile sites), which suggest that they are deeply involved in the development, metastasis, and recurrence of cancer (Calin et al., 2004). Lawrie et al. (2007) reported that a quantity of miRNA have been detected in the human serum. When compared to healthy controls, miR-155, miR-210, and miR-21 were significantly upregulated in the serum of patients who suffered from diffuse large B-cell lymphoma, implicating the potential diagnostic efficacy of these serum miRNA. In recent years, several groups have identified miRNA in NSCLC tissue samples, and a number of promising studies detected changes in circulating miRNAs in blood samples of NSCLC patients. These results could potentially lead to the application of non-invasive detection methods as well as new molecular approaches to treating NSCLC, or even to monitor the efficacy of the therapy. The sequent research (Chen et al., 2008; Mitchell et al., 2008) uncovered the application of circulating miRNA, a novel, non-invasive biomarker which existed stably in the serum. Compared with human plasma, there exists no blood cells in human serum. There are other parameters that cannot be ignored in blood cells, which could confuse the results of detection once the blood cells were disrupted. Consequently, serum miRNA is more likely to be utilized as a dependable tumor biomarker than plasma miRNA. Lv.et al studied the serum miRNA profile of early lung adenocarcinoma (Lv et al., 2017), although non-adenocarcinoma should not be ignored. Therefore, our study aims to identify and verify the differentially expressed (DE) miRNAs in serum collected from NSCLC patients in early stage and healthy controls by real-time fluorescent quantitative PCR (qRT-PCR). We discovered potential biomarkers for early diagnosis of NSCLC patients with a view to improving the diagnosis rate, promoting prognosis, and reducing mortality.

## Materials and Methods

### Study Population

All the serum samples came from patients who were newly diagnosed in the department of cardiothoracic surgery of Nanfang Hospital and from healthy age- and gender-matched individuals at the Nanfang Hospital Health Checkup Center from the period of January 1, 2014 to June 1, 2016. None of the Stage I or II patients (according to the 2009 AJCC/UICC 7th Edition TNM staging) had received any anti-tumor treatments including surgery, radiotherapy, or chemotherapy. Their clinical characteristics are listed in Supplementary Tables 1–3. All patients and healthy controls were informed by the full-time nurses of the main purpose, basic procedures, potential risks, and clinical significance of this study. The written informed consent form was signed by the patients or the healthy individuals. Our experiment was reviewed and approved by the Nanfang Hospital Ethics Committee of Southern Medical University.

### Sample Processing

Serum samples were collected from all the participants’ venous blood. Samples were centrifuged at 1,500 rpm for 15 min, then stored in -80°C for further analysis. The total RNA was extracted from the serum samples by using Tizol Reagent (BD Molecular Research Center, United States) according to the protocols of the manufacturers. In accordance with the instructions of the manufacturers, miRNA labeling and hybridization were conducted by using miRCURY^TM^ Array Power Labeling kit (Exiqon, Denmark, 208032-A) and miRCURYTM Array Wash buffer kit (Exiqon, Denmark, 208021).

### miRNA Chip Scanning and Data Analysis

Chip screening for fluorescence intensity was performed by using Axon GenePix 4000B Microarray scanner (Molecular Devices, United States) to convert images into digital signals. GenePix V6.0 was used to read the original fluorescence intensity of each probe in the chip. A total of 3,100 miRNAs were measured. The correction value was obtained by subtracting the background value from the original value and averaging the values of the four repeated points of each probe point on the same chip. miRNAs with significant differential expression were determined by T test with a difference ≥2 times.

### miRNA Reverse Transcription Reaction and RT-PCR

Reverse transcription reaction of the candidate serum miRNA (miR-185-5p, miR-431-5p, miR-484, miR-492, miR-584-5p, miR-590-3p, and miR-631) was performed by using PrimeScript^TM^ RT reagent Kit (Takara, Dalian, China, RR037A) according to the manufacturer’s instructions. RT-PCR was carried out with SYBR^®^ Premix Ex Taq^TM^ II (Tli RNaseH Plus) (Takara, Dalian, China, RR820A) in accordance with the manufacturer’s protocols. The relative expressions of the candidate miRNA were calculated by 2^–ΔΔCT^. Primers of reverse transcription and RT-PCRs were acquired from Guangzhou Ribo Bio Inc (China, Guangzhou). U6 was considered as the endogenous control.

### Receiver Operating Characteristic Analysis of the Differentially-Expressed miRNA

The receiver operating characteristic (ROC) curve, as a comprehensive index which reflects the sensitivity and specificity of continuous variants, can be used in the evaluation of the diagnostic value of the diagnostic test. When comparing the diagnostic value of two or more different diagnostic tests, the area under the ROC curve (AUC) of each test was calculated for direct comparison. The ROC curves of serum DE-miRNAs were drawn, respectively.

### Gene Expression Omnibus Database

Gene expression data of paired NSCLC patients’ cancer tissues (tumor tissues and para-cancerous tissues) and healthy human serum were obtained from Gene Expression Omnibus (GEO)^1^ database, and screened for differentially expressed miRNAs with a *p*-value less than 0.05. Studies were regarded eligible for our analysis according to the following criteria: (1) Studies with non-small cell lung cancer tissue or serum samples, (2) Studies with information about the technology utilized, and (3) Studies with the existence of normal cohorts as the control. Based on these rules, five datasets were obtained from the GEO database, including tumor tissues from non-small lung cancer patients and adjacent tissues (GSE2109, GSE74190, and GSE63805), and serum from lung cancer patients and healthy controls (GSE64591 and GSE20189). Next, we performed the differential analysis (|Log_2_FC| > 2, *p*-value < 0.05) by comparing tumor tissues to normal tissues in the SPSS 19.0 software using student’s *t*-test. Subsequently, we combined the differentially expressed genes acquired from GEO and gene chips to get the shared gene signatures.

### Prediction of Target Genes of the DE-miRNAs

The V7.0 miRTarBase,^2^ which is an online database containing plenty of experimentally validated microRNA–target interactions (Hsu et al., 2011), was applied to identify the potential target genes of the DE-miRNA. Species selects: *Homo sapiens*. miRNA ID was entered as “hsa-miR-590-3p/hsa-miR-492/hsa-miR-631” to obtain the gene list of predicted target genes. Since the list contains a small number of strong evidence genes, we selected all the genes in the gene list (including strong evidence and less strong evidence) for relevant pathway enrichment analysis.

### GO and KEGG Pathway Analysis

The DAVID database (version 6.8),^3^ as a bioinformatic database that integrates biological data and analysis tools to provide systematic and comprehensive biological annotation information for large-scale gene or protein lists, was used to perform gene enrichment analysis of the collection of the potential target genes of the DE-miRNAs (Dennis Sherman et al., 2003). We have taken a collection of the predicted target genes of the three target miRNAs respectively to obtain the gene list. On the background of the human genome-wide annotation data (org.Hs.eg.db), we utilized the cluster profiler package of the R language (version: 3.6.5) to perform the enrichment analysis of GO and KEGG pathways. The *p*-value significance threshold is set to 0.5, and the *q*-value significance threshold is set to 0.2. GO functional annotation analysis and KEGG pathway analysis based on the DAVID database were conducted on the candidate target genes of the DE-miRNAs. *P*-value < 0.05 was regarded as statistically significant.

### Statistical Analysis

Data analysis was performed by SPSS 19.0 (Chicago, IL, United States) software and measurement data was expressed by mean ± standard deviation. Two independent samples T test and χ^2^ Test were performed to compare differences in miRNA expression between two groups. Mann–Whitney tests were used to analyze differential expression; *p*-value less than 0.05 was regarded as statistically significant. ROC curves were established to analyze the diagnostic effects of serum miRNAs and the AUC calculated their specificity and sensitivity in the diagnosis of early NSCLC. In terms of prediction of diagnostic efficacy of combination of multiple serum miRNAs, the process was followed by setting up regression models by using binary logistic regression method.

## Results

### Screening for Differentially Expressed Serum miRNAs by Chip and Bioinformatics Analysis

The serum total RNA samples were extracted from six patients with early NSCLC and six healthy controls. Each sample was made into one gene chip and then a total of 12 gene chips were screened for the differentially expressed miRNAs. It was shown that 209 serum miRNAs exhibited a differential expression of two-fold or more, of which 122 miRNAs were up-regulated and 87 miRNAs were down-regulated. Then we downloaded a data set containing information about gene expression of NSCLC patients and healthy human serum, as well as NSCLC patients and para-cancerous tissues from GEO, screening for differentially expressed miRNAs with P value less than 0.05. The expression levels of 31 miRNAs were significantly up-regulated in NSCLC patients compared to healthy individuals, meanwhile 28 miRNAs were obviously down-regulated in serum samples. As for samples from tissues, 309 miRNAs were found to express differentially in NSCLC and adjacent tissues, in which 172 miRNAs were significantly up-regulated and 137 miRNAs were down-regulated. With a view of further enhancing the reliability of the chip results, we crossed the differentially expressed miRNAs screened by gene chip with that obtained by data mining. The miR-484 and miR-590-3p were significantly up-regulated. When intersecting the data-set results from tissues and that of gene chips, five miRNAs were expressed differentially, including up-regulated miR-185-5p, miR-431-5p, and miR-492 and down-regulated miR-584-5p and miR-631 (Table 1).

**TABLE 1:** The differentially expressed miRNAs initially screened by gene chip and data mining.

miRNA	Gene chip		Data Mining		
		
	Fold change	*p* value	Sample source	Fold change	*p* value
miR-185-5p	3.488	0.002	Tissue	1.111	0.003
miR-431-5p	2.707	0.011	Tissue	4.377	0.018
miR-484	2.253	0.037	Serum	2.253	0.037
miR-492	3.882	0.021	Tissue	2.997	0.003
miR-584-5p	0.184	0.005	Tissue	0.708	0.000
miR-590-3p	3.534	0.015	Serum	3.534	0.015
miR-631	0.222	0.010	Tissue	0.564	0.009

### Candidate Serum miRNAs Screened From Clinical Serum Samples

**FIGURE 1:**
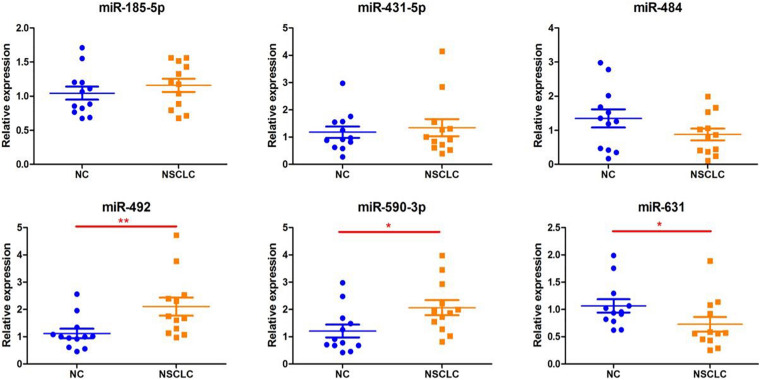
Differentially expressed serum miRNAs in the training set. The miRNA levels of miR185-p, miR-431-5p, miR484, miR-492, miR-590-3p, and miR-631 were detected by RT-PCR assay. Data are presented as mean ± SD. *p < 0.05 vs. NC group, **p < 0.01 vs. NC group.

**FIGURE 2:**
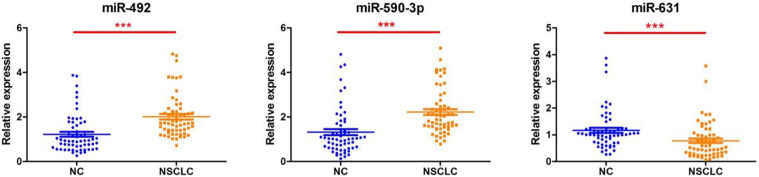
Differentially expressed serum miRNAs in the validation cohort. The miRNA levels of miR-492, miR-590-3p, and miR631 were detected by RT-PCR assay. Data are presented as mean ± SD. ***p < 0.001 vs. NC group.

We extracted serum total RNA from 24 cases in the training cohort, and seven candidate serum miRNAs obtained by initial screening were detected by RT-PCR. As shown in Figure 1, serum miR-492 and miR-590-3p of those who suffered early disease were significantly up-regulated compared to the healthy individuals (1.88 and 1.71 folds, P values were 0.008 and 0.0141, respectively). As shown in Figure 1, miR-631 was down-regulated 1.46 times (*p* = 0.0304). miR-185-5p, miR-431-5p, and miR-484 also presented differential expression without statistical difference. In addition, the Ct value of miR-584-5p was greater than 40, suggesting that the serum concentration of it is low. Serum total RNA were collected from 120 cases in the validation cohort, and RT-PCR was performed to further validate the three candidate miRNAs screen from the training cohort (Figure 2). Compared to healthy individuals, miR-492 and miR-590-3p in the patients’ serum were up-regulated 1.65 and 1.69 fold, respectively (*p* < 0.0001). It is suggested that the RNA expression levels of miR-492 and miR-590-3p were obviously up-regulated in early-stage NSCLC patients. The serum miR-631 was down-regulated 1.50 times (*p* < 0.0001).

### Diagnostic Efficacy of Differential Expression of miR-492, miR-590-3p, and miR-631

**FIGURE 3:**
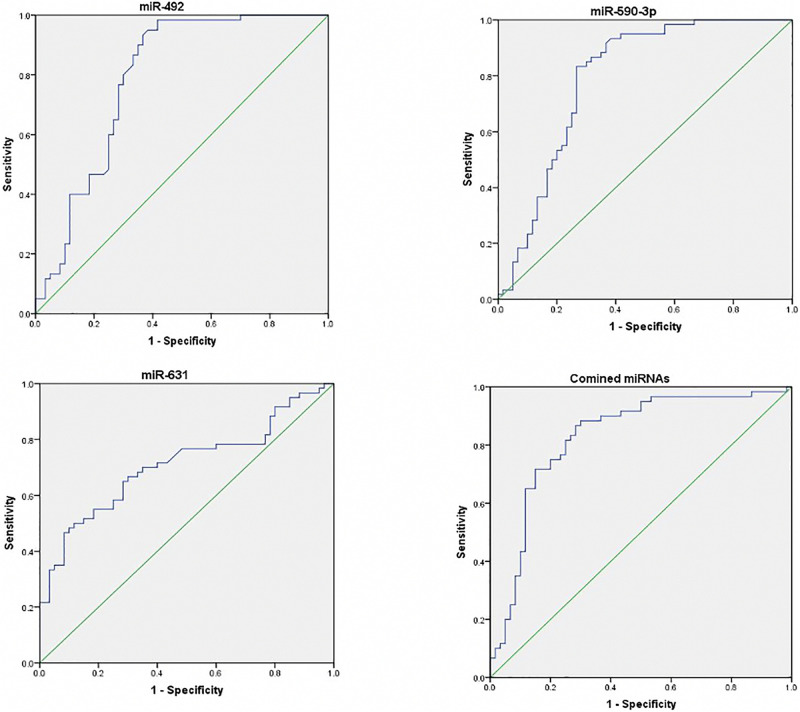
Receiver operating characteristic (ROC) curves of differentially expressed miRNAs between NSCLC patients and healthy controls. ROC curves of miR-492, miR-590-3p, and miR631 showed a moderate distinguishing efficiency. The combination of the three miRNAs showed a slightly higher AUC value.

The ROC curves of three serum miRNAs were drawn. The results showed that the AUC value of miR-492, miR-590-3p, and miR-631were 0.789, 0.792, and 0.711, respectively when distinguished between healthy individuals and early stage NSCLC patients. As illustrated in Figure 3, the AUC reached 0.828 (95% CI, 0.750–0.905, *p* = 0.000) when using these three serum miRNAs as a combination to discriminate healthy volunteers from patients.

### The Correlation of miR-492, miR-590-3p, and miR-631 With Clinicopathological Factors

**FIGURE 4:**
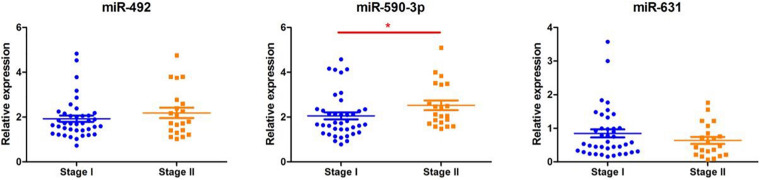
The relationship between miRNA expression levels in serum and with clinical stage. There were no significant differences between miR-492 or miR-631 expression levels in serum with clinical stage. The expression level of serum miR-590-3p increases with clinical stage. *p < 0.05.

**FIGURE 5:**
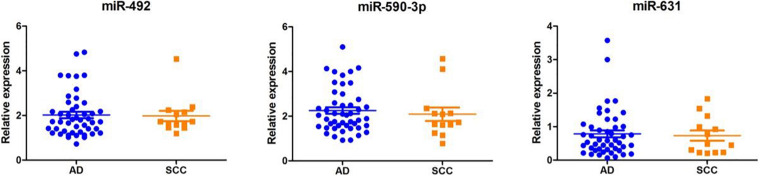
The relationship between miRNA expression levels in serum and with pathological types. Serum miR-492, miR-590-3p, and miR-631 miRNAs did not show significant differential expression in adenocarcinoma and squamous cell carcinoma.

As shown in Figure 4, according to the TNM staging classification, the expression levels of serum miR-492 and miR-631 did not significantly increase with the progress of tumor TNM staging (miR-492: *p* = 0.385, miR-631: *p* = 0.265). The expression of miR-590-3p in the serum of stage II patients was notably higher than stage I patients (*p* = 0.042). When classified by pathological type, serum miR-492, miR-590-3p, and miR-631 did not express in a significant difference in the adenocarcinoma and squamous cell carcinoma (miR-492: *p* = 0.781, miR-590-3p: *p* = 0.572, miR-631: *p* = 1.000) in Figure 5.

### The Target Genes of miR-492, miR-590-3p, and miR-631 Were Associated With Lung Cancer Progression

**FIGURE 6:**
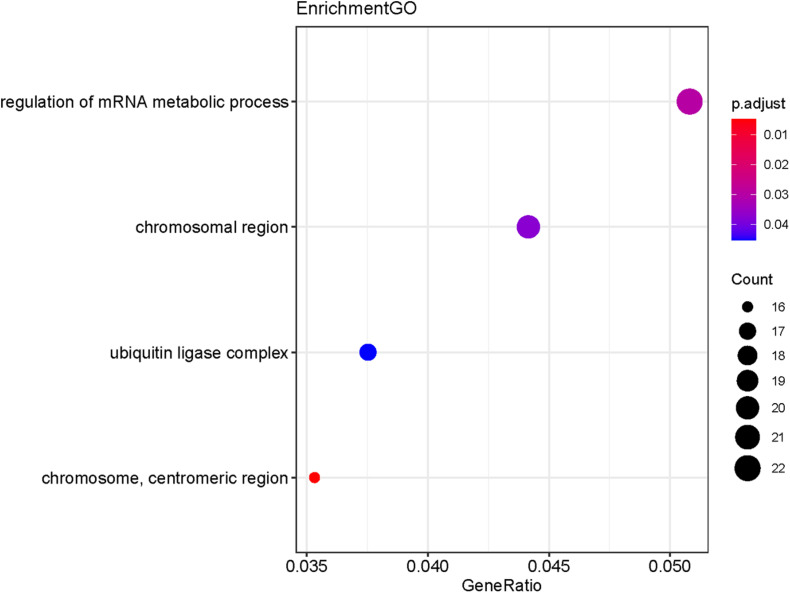
GO functional annotation of the collection of the potential target genes of the DE-miRNAs (miR-492, miR-590-3p, and miR-631). The functions of the target genes were mainly enriched in the regulation of mRNA metabolic process, chromosomal region, ubiquitin ligase complex and chromosome, and centrometric region. *P*-value < 0.05 was considered statistically significant.

**FIGURE 7:**
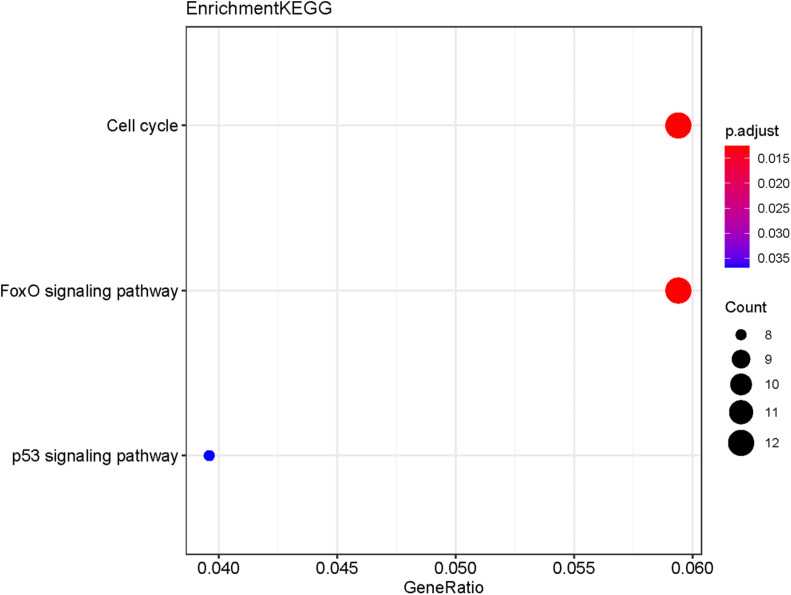
KEGG pathway analysis of the collection of the potential target genes of the DE-miRNAs (miR-492, miR-590-3p, and miR-631). The results show the enriched pathways of the target genes include cell cycle, FoxO signaling pathway, and P53 signaling pathway.

The potential target genes of the DE-miRNA, including miR-492, miR-590-3p, and miR-631, were predicted using the miRTarBase database. As shown in Supplementary Table 5, 45 target genes were predicted for miR-492, 390 genes for miR-590-3p, and 51 genes for miR-631. GO functional annotation analysis on the above-mentioned target genes was performed, as shown in Figure 6. The GO analysis of the collection of target genes of miR-492, miR-590-3p, and miR-631were enriched in the following functions, including the regulation of mRNA metabolic process, chromosomal region, ubiquitin ligase complex and chromosome, and centrometric region. Studies demonstrated that the genetic mutation of chromosomal regions were closely associated with tumorigenesis of lung cancer (Li et al., 2016). It was reported that various ubiquitin ligase complexes were related to cell stemness maintenance and cell migration of lung cancer cells (Shao et al., 2018; Gu et al., 2019). To further explore the potential enriched pathway relevant to these target genes, KEGG pathway analysis of the collection of the DE-miRNAs’ target gene was subsequently conducted using the 6.8 DAVID database. As shown in Figure 7, the differential target genes of miR-492, miR-590-3p, and miR-631 were enriched in cell cycle, FoxO signaling pathway, and P53 signaling pathway. P53 was a typical tumor suppressor gene. Several studies indicated that P53 mutation serves as a poor prognosis for NSCLC, particularly lung adenocarcinoma (Brambilla and Brambilla, 1997). Furthermore, P53 pathway provides some target for treatment of lung cancer (Mitsudomi et al., 1995). The expression of cell cycle was involved in a variety of genetic alterations in the process of pathogenesis and progression of both NSCLC and SCLC. It was reported that the forkhead box subfamily O (FOXO) pathway, which is downstream of the PI3K/AKT pathway, could promote the apoptosis of NSCLC cells.

## Discussion

A majority of all the newly diagnosed cases were at advanced stage or had developed distant metastasis (Reck and Rabe, 2017). Therefore, it is urgent to develop an early detection assay for NSCLC patients. Compared to tissue biopsy, blood is commonly used in clinical laboratory tests with a low cost which helps to reflect the physical condition in a more all-round way. It is also a more favorable non-invasive method which could be repeatedly tested. miRNAs were found to be able to exist steadily in the circulatory system. An increasing number of scientific researchers are focusing on circulatory microRNAs (Ntoumou et al., 2017; Van Roosbroeck and Calin, 2017; Huang et al., 2019) in the hope of their utilization in tumor diagnosis, prognosis, and efficacy assessments. This study was designed to find out new serum biomarkers of miRNA for early diagnosis of non-small cell lung cancer.

High-throughput gene chip was taken advantage of to screen differentially expressed serum miRNAs in patients with early-stage NSCLC. Then methodology of bioinformatics was performed for data mining, and we ultimately screened out seven potential serum miRNAs: miR-185-5p, miR-431-5p, miR-484, miR-492, miR-584-5p, miR-590-3p, and miR-63. Then we divided 144 patients into two cohorts: the training cohort and validation cohort. miR-492, miR-590-3p, and miR-631 were finally identified as the statistically different miRNAs. The ROC curve analysis showed that the sensitivity of a collection of these three serum miRNAs can reach up to 86.7%, and the specificity was as high as 71.7%, which indicated that they could be used as potential biomarkers in the early diagnosis of NSCLC.

It was reported in several studies that miR-492 was highly expressed in many different tumors and was associated with the process of tumorigenesis, metastasis, and recurrence (von Frowein et al., 2018; Shi et al., 2019). Using the technology of gene chips, Wang et al. (2019) aimed to discover miRNA expression profiling of liver cancer and its adjacent tissues and found that miR-492 was highly expressed in liver cancer tissues. Furthermore, miR-492 was proven to be involved in the development of liver cancer by *in vitro* and *in vivo* experiments (Jiang et al., 2014). Shen et al. (2015) noted that the expression level of miR-492 was up-regulated both in breast cancer cells and tissues, which facilitates breast cancer cell proliferation and growth. Bioinformatic analysis and experimental researches show that SOX7 is a potential target gene of miR-492, and miR-492 could promote cell cycle of breast cancer cells by downregulating the expression of SOX7 (Wang et al., 2020). It was in accordance with the results of KEGG enrichment analysis indicating the target genes were enriched in cell cycle. In addition, it was reported that p53 activation is capable of upregulating miR-492 in lung cancer cells using a miRNA profiling assay (Shen et al., 2015). It was in agreement with the fact that KEGG analysis shows that target genes were enriched in P53 signaling pathway.

As far as miR-590-3p is concerned, it is not only highly expressed in lung adenocarcinoma tissues and metastatic lymph nodes, but is also expressed at a high level in lung adenocarcinoma cells. Moreover, investigators found that miR-590-3p inhibited the expression of OLFM4 protein by binding to the 3’UTR site of OLFM4, which is its downstream target gene, thus promoting invasion and metastasis of lung adenocarcinoma (Liu et al., 2017). In addition, Liu et al. found that the miR-590/Acvr2a/Terf1 signaling pathway exerts effects on modulating telomere elongation of induced pluripotent stem cells (Liu et al., 2018). And our results of GO analysis were enriched in the chromosomal region. Apart from that, relevant research demonstrated that hsa-miR-590-3p could serve as the potential regulator of COMMD10, whose target genes were dominantly enriched in Cullin-RING ubiquitin ligase complexes (Fan et al., 2020). What’s more, Shi et al. (2020) found that Hsa-MiR-590-3p could facilitate the progression of pancreatic cancer through G1/S cell cycle pathway. Abdolvahabi et al. showed that MiR-590-3p suppresses cell growth and promotes the apoptosis of breast cancer cell via deacetylation of p53 (Abdolvahabi et al., 2019). And the above-mentioned discoveries were in accordance with the results of our GO and KEGG analysis. GO functional annotation analysis using the DAVID database shows the collection of the potential target genes were enriched in ubiquitin ligase complex. KEGG enrichment analysis indicates enriched pathways in cell cycle and P53 signaling pathway.

Fu et al. (2016) detected that miR-631 was down-regulated in prostate cancer cells and tissues. Meanwhile, it was found that miR-631 was related to patients’ drug resistance to bortezomib in multiple myeloma. Xi et al. (2017) found that UbcH10 was highly expressed in bortezomib-resistant multiple myeloma, which was caused by low expression of miR-631. The results of the above basic research revealed molecular mechanisms of three miRNAs with tumorigenesis. We believe that the above miRNAs may be used as potential biomarkers with an important diagnostic significance for early diagnosis of NSCLC. Targeting these enriched pathways could contribute to the comprehension of how miR-492, miR-590-3p, and miR-631 regulate the process of pathogenesis and progression of NSCLC.

Compared to previous studies with regard to serum miRNAs, our study has several advantages. On the one hand, in the initial screening stage of the target gene, in addition to the chip technology that was often applied in high-throughput screening of hallmarks, we also adopted the popular bioinformatic method for data mining so as to enlarge sample size and enhance the reliability of our results. On the other hand, studies prior to this experiment have generally not restricted the tumor staging in the population and incorporated a considerable number of stage III or IV NSCLC. However, all subjects included in our study were early-stage NSCLC patients with a better survival prognosis. Therefore, it is more imperative for us to explore potential biomarkers for early diagnosis of these patients. What’s more, the enriched functions and pathways in GO and KEGG analysis conducted on the collections of the potential target genes could facilitate a comprehensive understanding of pathways influenced by miR-492, miR-590-3p, and miR-631.

In summary, our study identified a diagnostic biomarker consisting of three serum miRNAs (miR-492, miR-590-3p, and miR-631) for early NSCLC, which has certain clinical significance. Although the sample number is not large, the conclusion from the preliminary exploration was almost in accordance with the results of the validation set. Therefore, it is urgent to expand the sample size in the future, and further work is needed to validate these findings in a larger cohort and in the prospective setting as an actionable biomarker. Focusing on the enriched pathways such as cell cycle, FoxO signaling pathway, and P53 signaling pathway could further contribute to the understanding of the underlying mechanisms of how miR-492, miR-590-3p, and miR-631 impact on tumorigenesis and progression of lung tumor.

## Data Availability Statement

The datasets presented in this study can be found in online repositories. The names of the repository/repositories and accession number(s) can be found below: https://www.ncbi.nlm.nih.gov/geo/query/acc.cgi?acc=GSE171517.

## Ethics Statement

The studies involving human participants were reviewed and approved by the Nanfang Hospital Ethics Committee of Southern Medical University. The patients/participants provided their written informed consent to participate in this study. Written informed consent was obtained from the individual(s) for the publication of any potentially identifiable images or data included in this article.

## Author Contributions

XD, DL, and SQ: formal analysis, manuscript preparation, and writing. XZ: conception of the study, academic instruction, funding acquisition, constructive discussions, manuscript reviewing, and editing. XD, ZZ, and QW: data extraction, data curation, resources, and software. All authors contributed to the article and approved the submitted version.

## Conflict of Interest

The authors declare that the research was conducted in the absence of any commercial or financial relationships that could be construed as a potential conflict of interest.

## Footnotes

1 http://www.ncbi.nlm.nih.gov/geo/

2 http://miRTarBase.cuhk.edu.cn/

3 http://david-d.ncifcrf.gov/
