# Supplementary material for: Circulating miRNAs in Serum as Biomarkers for Early Diagnosis of Non-small Cell Lung Cancer
Source: Front Genet. 2021 Jul 9;12:673926. doi: 10.3389/fgene.2021.673926 (PMC8299278; doi:10.3389/fgene.2021.673926)
Supplement: Supplementary file 1 [file Data_Sheet_1.docx]

Supplement material.

**Table S1.** Clinical characteristics of 6 patients with lung cancer.

| Name | Gender | Age | Pathology | TNM | Stage |
| --- | --- | --- | --- | --- | --- |
| **DSX** | Male | 62 | SCC | T2aN0M0 | IB |
| **LXD** | Female | 60 | SCC | T2aN0M0 | IB |
| **FLC** | Male | 61 | SCC | T3N0M0 | IIB |
| **ZFK** | Male | 55 | AD | T1aN0M0 | IA |
| **OYKC** | Male | 69 | AD | T1bN0M0 | IA |
| **HYC** | Female | 67 | AD | T2aN0M0 | IB |

Abbreviation: SCC: squamous cancer cell; AD: adenocarcinoma.

**Table S2.** Clinical characteristics of 12 patients with lung cancer and 12 healthy controls.

|  | **Healthy people** | **Lung cancer patients** | **P value** |
| --- | --- | --- | --- |
| **Age** | 50.42 ± 4.01 | 52.83 ± 10.69 | 0.47 |
| **Gender** |  | | |
| **Male** | 7 | 6 | 1.00 |
| **Female** | 5 | 6 |  |
| **Stage** |  | | |
| **I** | - | 8 |  |
| **II** | - | 4 |  |
| **Pathology** |  | | |
| **AD** | - | 8 |  |
| **SCC** | - | 4 |  |

Abbreviation: SCC: squamous cancer cell; AD: adenocarcinoma.

**Table S3.** Clinical characteristics of 60 patients with lung cancer and 60 healthy controls.

|  | **Healthy people** | **Lung cancer patients** | **P value** |
| --- | --- | --- | --- |
| **Age** | 50.62 ± 5.65 | 52.72 ± 9.16 | 0.13 |
| **Gender** |  | | |
| **Male** | 32 | 36 | 0.58 |
| **Female** | 28 | 24 |  |
| **Stage** |  | | |
| **I** | - | 39 |  |
| **II** | - | 21 |  |
| **Pathology** |  | | |
| **AD** | - | 47 |  |
| **SCC** | - | 13 |  |

Abbreviation: SCC: squamous cancer cell; AD: adenocarcinoma.

**Table S4.** Results of total RNA from quality analysis

| Sample | **OD260/280** | **OD260/30** | **concentration（ng/μL）** | **Volume（μL）** | Quality**（ng）** |
| --- | --- | --- | --- | --- | --- |
| **DSX** | 1.56 | 0.63 | 26.55 | 10 | 265.50 |
| **LXD** | 1.24 | 0.37 | 31.27 | 10 | 312.70 |
| **FLC** | 1.31 | 0.55 | 30.20 | 10 | 302.00 |
| **ZFK** | 1.45 | 0.70 | 29.88 | 10 | 298.80 |
| **OYKC** | 1.34 | 0.53 | 30.66 | 10 | 306.60 |
| **HYC** | 1.36 | 0.26 | 29.93 | 10 | 299.30 |
| **N0050** | 1.35 | 0.51 | 31.12 | 10 | 311.20 |
| **N0082** | 0.99 | 0.27 | 35.77 | 10 | 357.70 |
| **N0085** | 1.23 | 0.29 | 23.79 | 10 | 237.90 |
| **N0032** | 1.43 | 0.73 | 31.79 | 10 | 317.90 |
| **N0065** | 1.50 | 0.31 | 26.82 | 10 | 268.20 |
| **N0098** | 1.34 | 0.64 | 25.70 | 10 | 257.00 |
